# Editorial Note: Design and characterization of an 87k SNP genotyping array for Arctic charr (*Salvelinus alpinus*)

**DOI:** 10.1371/journal.pone.0304369

**Published:** 2024-06-04

**Authors:** The PLOS ONE Editors

This article [1] cites a *PLOS ONE* article that was retracted on February 9, 2021 [2, 3] because the sample used for genome analysis was found to have been misidentified as it may have been from a hybrid between Arctic charr and a related species, Dolly Varden. The genomic assembly reported in [2] is still available at GenBank, but it has been relabeled as an unclassified species in the *Salvelinus* genus, or *Salvelinus sp*. (GenBank accession: GCF_002910315.2). The draft *Salvelinus sp*. genome was used for the determination of SNP location for the array in [1].

Of the 15 sources of fish used in the design and testing of the genotyping array (Table 1 in [1]), the authors are confident that those involving Fraser, Nauyuk and Icelandic populations are pure Arctic charr based on related research [4, 5] and geographical isolation of the populations from other species involved in potential hybridization. The Tree River strain is represented in 3 sources used in [1], and it is possible that these samples are hybrids of Arctic charr and Dolly Varden. The authors noted that this was previously discussed in [4].

A member of the Editorial Board advised that the SNP locations in the array may be approximations due to the misidentification of the sample used for the draft genome [2] and that the resolution of the array may be overrepresented due to the potential inclusion of Dolly Varden SNPs via the Tree River samples. They advised that a draft assembly using a confirmed pure Arctic charr sample could be used to evaluate the performance of the array.

The corresponding author agreed that an array based on only Arctic charr SNPs would provide higher resolution, and stated that subsequent analysis published in a PhD thesis [6] shows that the *Salvelinus sp* genome was useful as a reference for the location of SNPs within the Arctic charr genome. A second member of the Editorial Board indicated that the thesis supports these claims, and agreed with the comments of the first Editorial Board member.

The *PLOS ONE* Editors issue this Editorial Note to notify readers of the use of a potentially hybrid strain in the design of this array, and to provide a summary of the discussion regarding the potential implication of this on the efficacy and accuracy of the array.
